# Anthocyanin Oligomers Induce Apoptosis and Autophagy by Inhibiting the mTOR Signaling Pathway in Human Breast Cancer Cells

**DOI:** 10.3390/ph17010024

**Published:** 2023-12-22

**Authors:** Min-Gu Lee, Hyun-Jin Hong, Kyung-Soo Nam

**Affiliations:** Department of Pharmacology and Intractable Disease Research Center, School of Medicine, Dongguk University, Gyeongju 38066, Republic of Korea; mklee@dongguk.ac.kr (M.-G.L.); hyunjin5886@naver.com (H.-J.H.)

**Keywords:** anthocyanin oligomers, breast cancer cells, apoptosis, autophagy

## Abstract

Anthocyanin oligomers (AOs) are phytochemicals synthesized by fermenting anthocyanins extracted from grape skins and are more biologically active than monomeric anthocyanins. In this study, we evaluate the effects of an AO on triple-negative MDA-MB-231 and HER2-overexpressing SK-BR-3 breast cancer cells. The cell viability of MDA-MB-231 and SK-BR-3 cells was significantly inhibited in a concentration-dependent manner by AO treatment for 24 h, while delphinidin (a monomeric anthocyanin) had no effect on cell viability. In addition, the AO increased H2A.X phosphorylation (a marker of DNA damage), reduced RAD51 (a DNA repair protein) and survivin (a cell survival factor) protein levels, and induced apoptosis by caspase-3-dependent PARP1 cleavage in both cell lines. Surprisingly, the AO induced autophagy by increasing intracellular LC3-II puncta and LC3-II and p62 protein levels. In addition, the AO inhibited the mTOR pathway in MDA-MB-231 and SK-BR-3 cells by suppressing the HER2, EGFR1, and AKT pathways. These results demonstrate that the anti-cancer effect of the AO was due to the induction of apoptosis and autophagy via cleaved caspase-3-mediated PARP1 cleavage and mTOR pathway inhibition, respectively. Furthermore, our results suggest that anthocyanin oligomers could be considered potential candidates for breast cancer treatment.

## 1. Introduction

Breast cancer is responsible for the deaths of many women worldwide [1], and despite the options of chemotherapy, surgery, and radiation therapy, mortality rates remain high. Breast cancer types are broadly categorized as human epidermal growth factor receptor-2 (HER2)-overexpressing, estrogen receptor (ER)-positive, progesterone receptor (PR)-positive, or triple-negative breast cancer (TNBC), and of these, HER2-overexpressing and TNBC have the poorest prognoses. HER2-overexpressing breast cancer is effectively treated with trastuzumab, a targeted therapy that specifically inhibits the HER2 gene [2]. On the other hand, TNBC is a highly metastatic malignant form that has no targeted therapy because it is negative for all three markers, and therefore it is treated using conventional chemotherapy [3,4]. However, the toxicities of chemotherapeutics result in significant side effects, and thus, effective low-toxicity treatments with minimal side effects are required.

Recently, several research groups have focused on the ability of phytochemicals extracted from natural sources, such as fruits, vegetables, and other plants, to treat cancer. Phytochemicals have different biological actions, such as anti-inflammatory, antioxidant, and anti-cancer activities, and are non-toxic [5]. Anthocyanins are phytochemicals found in fruits and vegetables like grapes, blueberries, and black rice with diverse pharmacological activities, which include anti-cancer, anti-viral, and neuroprotective effects [6].

**Figure 1:**
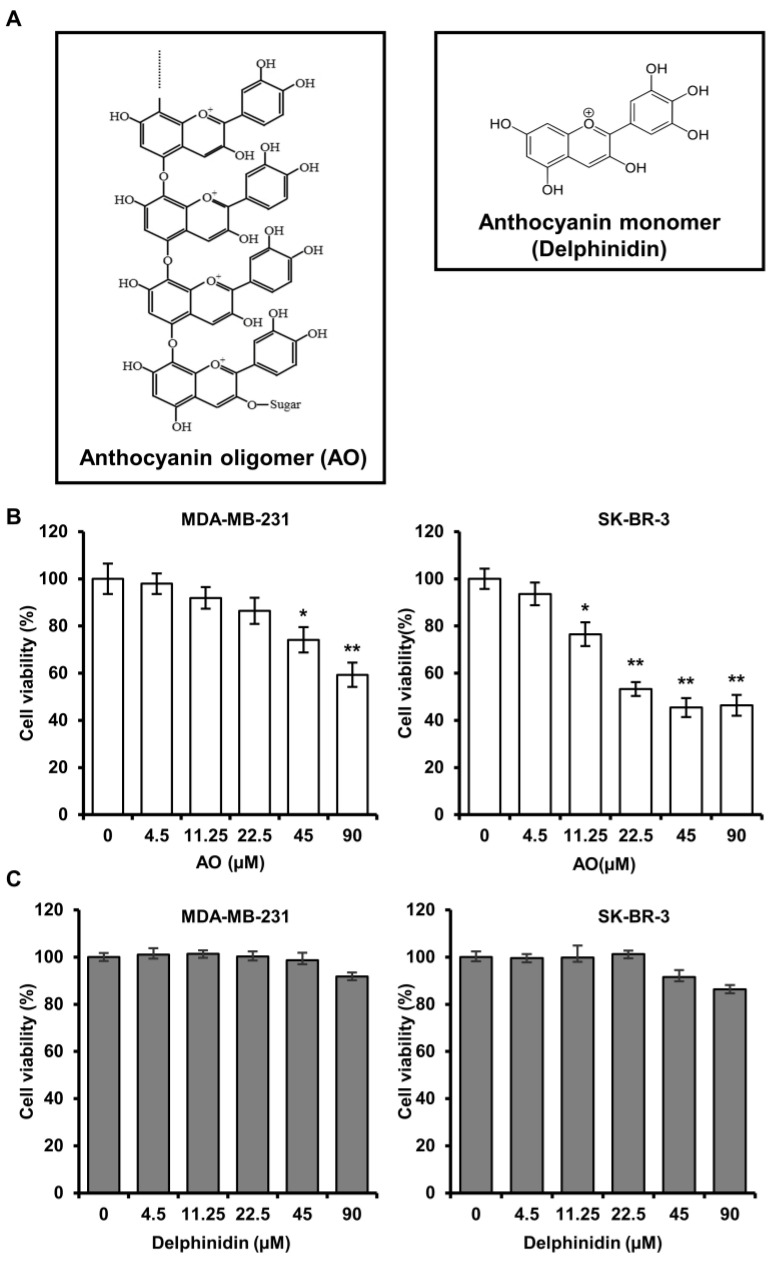
Comparative evaluation of the structure of AO and delphinidin (an anthocyanin monomer) and their effect on the cell viability in breast cancer cells. (**A**) Chemical structures of AO and delphinidin. MDA-MB-231 and SK-BR-3 cells were exposed to different concentrations of AO (**B**) and (**C**) delphinidin (0, 4.5, 11.25, 22.5, 45, 90 µM) for 24 h; cell viabilities were assessed using the SRB assay. Results are presented as means ± SDs (n = 8). Statistical significance is indicated by * representing *p*-values was less than 0.05, while ** represents was less than 0.01. The Statistical differences were assessed using one-way analysis of variance (ANOVA) and subsequently verified using Fisher’s least significant difference (LSD) test.

Anthocyanin oligomers (AOs) are polymeric forms of anthocyanin, whereby multiple anthocyanin monomers are connected to produce ring-like structures. AOs can be extracted from various plants, such as grapes (seed and skin) and blueberries but can also be synthesized chemically or enzymatically. According to a previous study, an AO produced by the *Aspergillus niger*-based fermentation of grape skin extract was more biologically active than the monomeric anthocyanin (Figure 1A) [7]. Furthermore, it has been reported that the antioxidant activity of this AO mixture improved symptoms in a mouse model of dry eye disease and promoted the browning of white adipocytes by increasing fat metabolism [8,9,10]. However, though much research has been conducted on the anti-cancer effects of plant-sourced AOs, comparatively little is known about the anti-cancer effects of AOs synthesized by fermentation. Therefore, this study aimed to assess the potential anti-cancer effect of AOs on breast cancer cells.

## 2. Results

### 2.1. AO Significantly Induced the Cytotoxicity of MDA-MB-231 and SK-BR-3 Cells

We performed an SRB assay to evaluate the efficacy of an AO (at 4.5, 11.25, 22.5, 45, and 90 µM) on cell viability for 24 h in MDA-MB-231 and SK-BR-3. The results showed that treatment with the AO (90 µM) significantly reduced cell viability to 59% and 46%, respectively (Figure 1B). Additionally, the AO also significantly reduced cell viability in a time- and concentration-dependent manner after treatment for 48 and 72 h (Figure S1). Furthermore, we performed an SRB assay under the same concentration conditions of the AO to compare and investigate the efficacy of delphinidin, a monomeric anthocyanin, on cell viability. Delphinidin did not significantly affect the cell viability of breast cancer cells (Figure 1C). Therefore, we demonstrated that the AO exhibits higher cytotoxic efficacy in breast cancer cells than monomeric anthocyanin. Since delphinidin showed no cytotoxic effects, all subsequent experiments were exclusively conducted using the AO.

### 2.2. AO Caused DNA Damage and Attenuated DNA Repair in MDA-MB-231 and SK-BR-3 Cells

**Figure 2:**
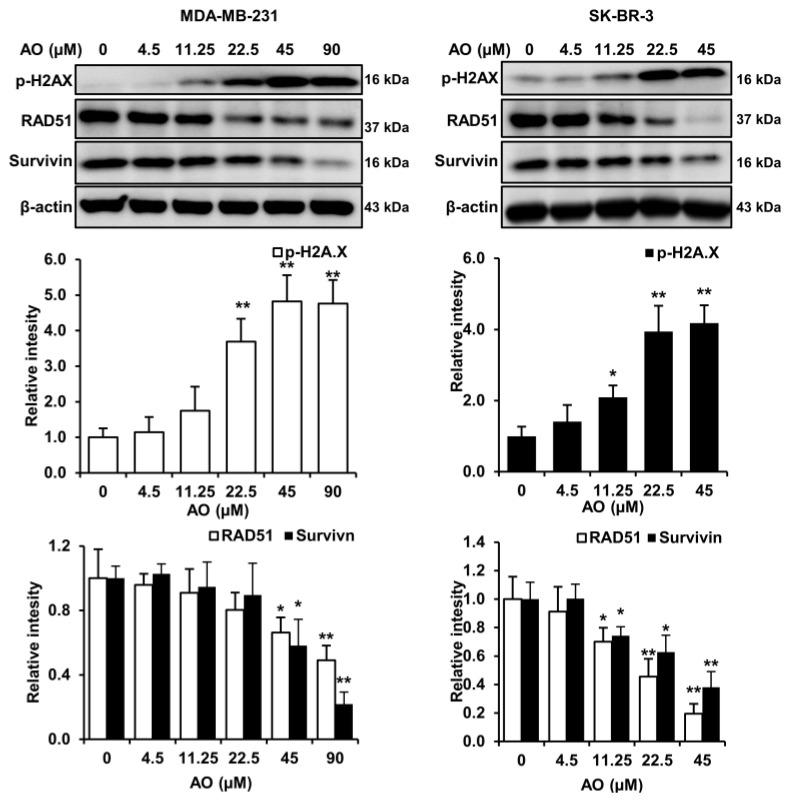
Effect of AO on DNA damage and DNA repair-related proteins in MDA-MB-231 and SK-BR-3 cells. Western blot analysis of phosphorylated H2A.X, RAD51, and survivin levels in breast cancer cells. Differences in relative intensities of protein bands were measured using ImageJ software (version 1.6.0_20). The Bands were normalized to β-Actin. Results are presented as means ± SDs (n = 3). Statistical significance is indicated by * representing *p*-values was less than 0.05, while ** represents was less than 0.01. The Bands were normalized to β-Actin. The Statistical differences were assessed using one-way ANOVA and subsequently verified using Fisher’s LSD test.

Generally, cytotoxic substances induce cell death by causing DNA damage and inhibiting DNA repair. Therefore, we analyzed changes in markers related to DNA damage and repair in MDA-MB-231 and SK-BR-3 cells treated with the AO for 24 h. The AO significantly elevated H2A.X phosphorylation (a DNA damage marker) and significantly suppressed levels of survivin and RAD51, which play crucial roles in DNA repair, in both cell lines (Figure 2). These results suggest that the AO induced DNA damage and inhibited DNA repair in SK-BR-3 and MDA-MB-231 cells.

### 2.3. AO Induced Apoptosis via Caspase-3 Dependent PARP1 Cleavage in MDA-MB-231 and SK-BR-3 Cells

**Figure 3:**
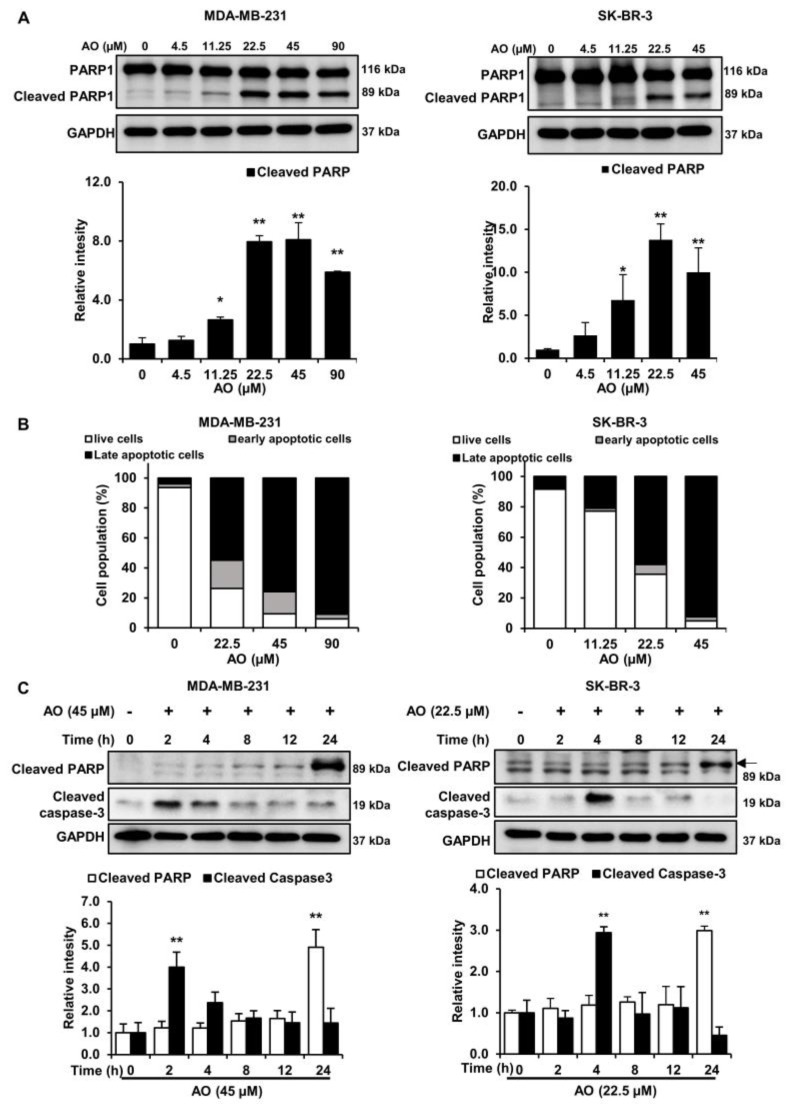
Effect of AO on apoptosis-related proteins and 7-AAD and annexin V staining in MDA-MB-231 and SK-BR-3 cells. (**A**) Changes in the expressions of PARP1 and cleaved PARP1 in breast cancer cells after 24 h of AO treatment. (**B**) Analysis of the effects of AO on apoptosis in MDA-MB-231 and SK-BR-3 cells using Annexin V/7-Aminoactinomycin D (7-AAD) staining. (**C**) Changes in the expressions of cleaved caspase-3 after 0, 2, 4, 8, 12, or 24 h of AO treatment. All band intensities were quantified using ImageJ software. The Bands were normalized to GAPDH. Results are presented as means ± SDs (n = 3). Statistical significance is indicated by * representing *p*-values was less than 0.05, while ** represents was less than 0.01. The statistical differences were assessed using one-way ANOVA and subsequently verified using Fisher’s LSD test.

To investigate the effect of the AO on the protein expressions of apoptosis-related factors in MDA-MB-231 and SK-BR-3, cells were treated with the AO (0–90 and 0–45 µM), respectively, for 24 h. We found that the AO increased PARP1 cleavage (89 kDa) in both cells (Figure 3A). Additionally, AO treatment increased in the late apoptotic cell population (Figure 3B). Despite the concentration-dependent increase in PARP cleavage and apoptosis, caspase-3 cleavage was not detected in either breast cancer cell 24 h after AO treatments. However, we obtained interesting results by analyzing the time course of PARP cleavage and caspase activation in response to AO treatment. Surprisingly, the cleavage of caspase-3 was observed in MDA-MB-231 and SK-BR-3 cells only after 2 and 4 h of AO treatment, respectively, while the cleavage of PARP increased only after 24 h of AO treatment in both cell lines (Figure 3C). Therefore, our results suggest that AO treatment leads to caspase-3 activation at the early stages, ultimately resulting in the cleavage of PARP.

### 2.4. AO Significantly Increased LC3-II and p62 Levels in the Two Cell Lines

**Figure 4:**
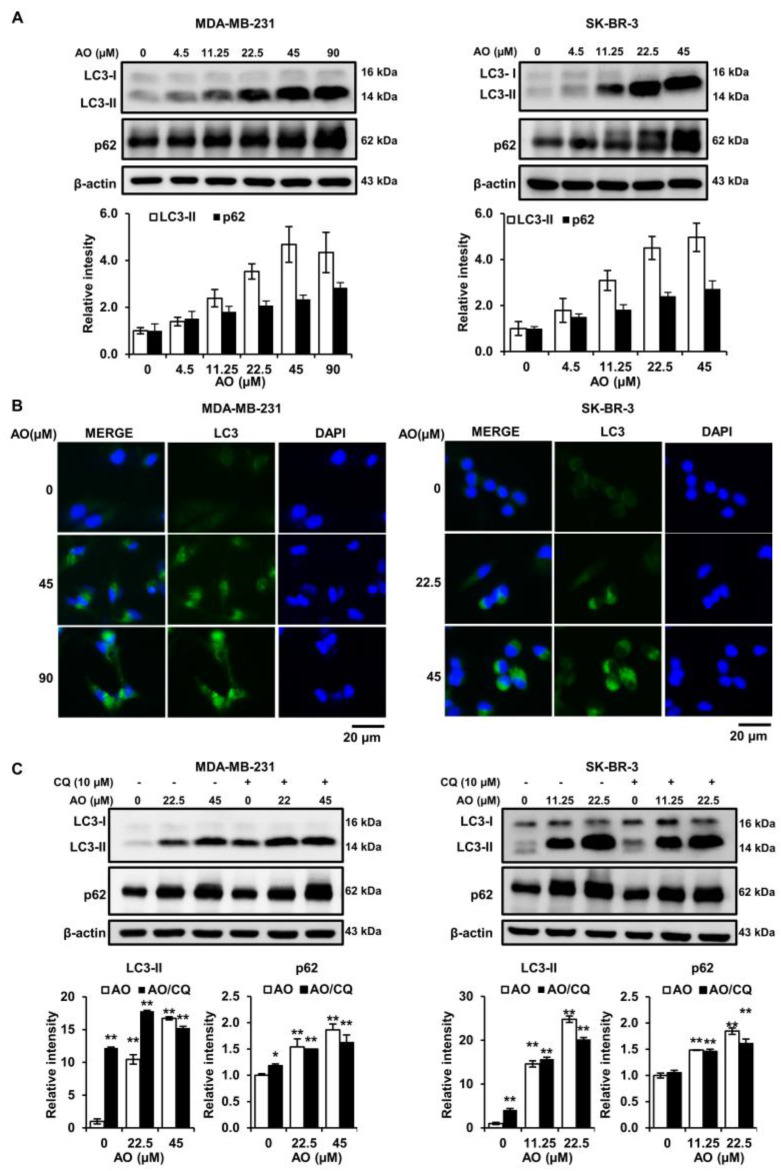
Effect of AO on autophagy-related proteins in MDA-MB-231 and SK-BR-3 cells. (**A**) Changes in level of LC3 and p62 proteins in breast cancer cells after 24 h of AO treatment. (**B**) Fluorescence microscopy images of LC3 puncta formation in breast cancer cells. (**C**) Changes in protein levels of LC3 and p62 due to combined treatment of AO and CQ (10 µM) for 24 h in breast cancer cells. All Bands intensities were quantified using ImageJ software. The Bands were normalized to β-Actin. Results are presented as means ± SDs (n = 3). Statistical significance is indicated by * representing *p*-values was less than 0.05, while ** represents was less than 0.01. The statistical differences were assessed using one-way ANOVA and subsequently verified using Fisher’s LSD test.

The experiments above showed that the AO induces DNA damage, leading to apoptosis. However, it is also known that DNA damage can trigger other programmed cell death pathways, such as autophagy. Therefore, we evaluated the effect of the AO on the autophagy markers (LC3-II and p62). The results presented that the levels of LC3-II and p62 proteins increased in a concentration-dependent manner after AO treatment in MDA-MB-231 and SK-BR-3 cells (Figure 4A). Also, AO treatment increased LC3 puncta formation in the cytoplasm, as determined by immunofluorescence staining (Figure 4B). Therefore, our findings indicated that the AO induces autophagy through the elevation of LC3-II and p62. Additionally, we treated with a combination of chloroquine (CQ) and the AO to demonstrate that the AO induced autophagy in breast cancer cells. CQ is an autophagy inhibitor that prevents the fusion of autophagosomes and lysosomes during the autophagy process. The results showed that the combined treatment of chloroquine (CQ) and AO still maintained the increased p62 and LC3-II protein levels from AO treatment alone (Figure 4C). These results suggest that despite the inhibition of lysosome fusion by CQ, the accumulation of autophagosomes occurs. Therefore, it can be suggested that the AO induces the autophagic process by accumulating autophagosomes in breast cancer cells.

### 2.5. AO Inhibited mTOR Pathways in MDA-MB-231 and SK-BR-3 Cells

**Figure 5:**
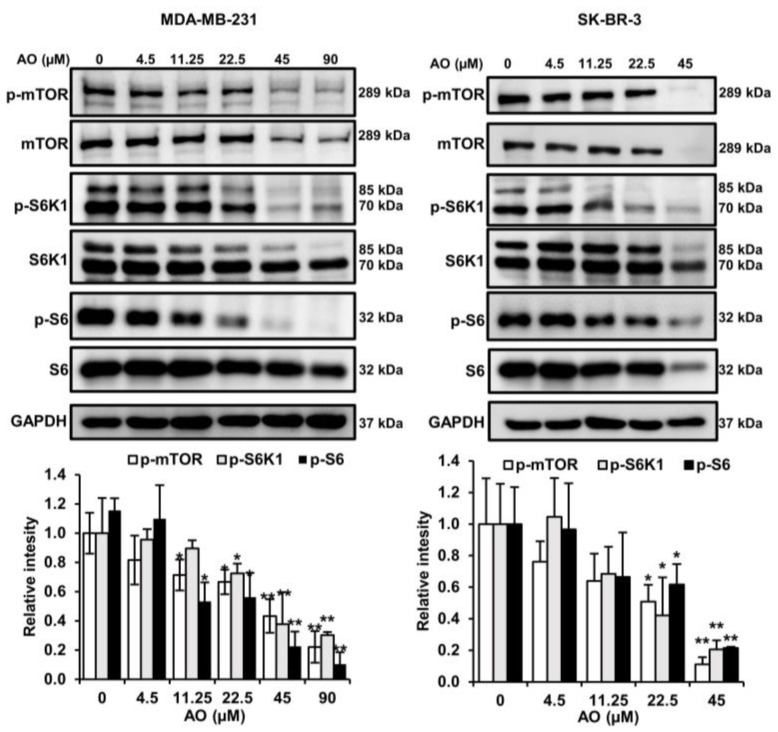
Effect of AO on the mTOR pathway. Changes in the protein and phosphorylation levels of mTOR, S6K1, and S6 after 24 h of AO treatment in MDA-MB-231 and SK-BR-3 cells. Band intensities were quantified using ImageJ software. The Bands were normalized to GAPDH. Results are presented as means ± SDs (n = 3). Statistical significance is indicated by * representing *p*-values was less than 0.05, while ** represents was less than 0.01. The statistical differences were assessed using one-way ANOVA and subsequently verified using Fisher’s LSD test.

LC3-II and p62 are regulated by the mTOR pathways, and thus, we analyzed the effect of the AO on the protein expressions and phosphorylation of mTOR in MDA-MB-231 and SK-BR-3. The AO significantly inhibited the protein and phosphorylation levels of mTOR, S6K1, and S6 in MDA-MB-231 and SK-BR-3 cells (Figure 5). Therefore, we found that the AO effectively inhibits the mTOR pathway. These findings suggest that the AO induced LC3II and p62 accumulation by suppressing the mTOR pathway in MDA-MB-231 and SK-BR-3 cells.

### 2.6. AO Inhibited the mTOR Pathway by Suppressing the HER2/EGFR1, and AKT Pathways in SK-BR-3 Cells

**Figure 6:**
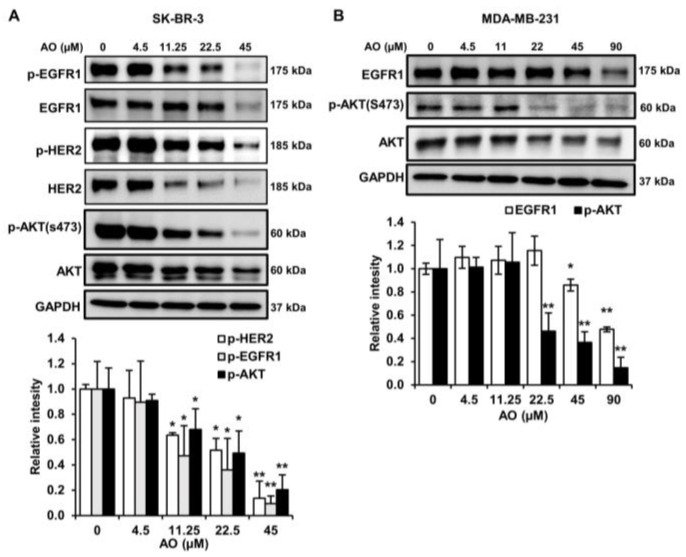
Effects of AO on HER2, EGFR1, and AKT pathways and EGFR1 and AKT pathways in SK-BR-3 and MDA-MB-231 cells, respectively. (**A**) Changes in the protein and phosphorylation levels of HER2, EGFR1, EGFR1, and AKT after treating SK-BR-3 cells with AO for 24 h. (**B**) Changes in the protein levels of EGFR1, p-AKT, and AKT after treating MDA-MB-231 cells with AO for 24 h. Band intensities were quantified using ImageJ software. The Bands were normalized to GAPDH. Results are presented as means ± SDs (n = 3). Statistical significance is indicated by * representing *p*-values was less than 0.05, while ** represents was less than 0.01. The statistical differences were assessed using one-way ANOVA and subsequently verified using Fisher’s LSD test.

We found that the AO inhibited the mTOR pathway in SK-BR-3 cells. We investigated the effect of the AO on the HER2, EGFR1, and AKT pathways. We found that the AO significantly suppressed the phosphorylation and protein levels of HER2 and EGFR1 and notably reduced the phosphorylation of AKT at s473, which lies downstream of HER2 and EGFR1 (Figure 6A). Hence, we suggest that the AO inhibits the mTOR pathway by attenuating the HER2, EGFR1, and AKT pathways in SK-BR-3 cells.

### 2.7. AO Inhibited the mTOR Pathway by Suppressing the EGFR1and AKT Pathways in MDA-MB-231 Cells

MDA-MB-231 is a TNBC cell line that lacks HER2 expression but expresses EGFR1. Thus, we examined the impact of the AO on EGFR1 expression in MDA-MB-231 cells. AO treatment reduced EGFR1 protein levels and effectively inhibited the phosphorylation of AKT (s473) (Figure 6B). These results suggest that the AO inhibits the EGFR1 and AKT signaling pathways in MDA-MB-231 cells and thus suppresses the mTOR pathway.

## 3. Discussion

Despite treatment advances, breast cancer continues to be a major cause of mortality among women. This study aimed to assess the anti-cancer effectiveness of a synthetic anthocyanin oligomer mixture (AO) and the mechanisms responsible for its effects in MDA-MB-231 and SK-BR-3 breast cancer cell lines.

Several studies have reported that the anti-cancer effects of natural anthocyanins on breast cancer cells are due to reduced viability via cytotoxicity [11,12]. Moreover, oligomeric anthocyanins are known to possess higher biological activity compared to monomeric anthocyanins [7,10,13]. Consistent with these findings, we found that a synthetic AO effectively reduced the cell viability of breast cancer cells compared to delphinidin (a monomeric anthocyanin) (Figure 1B,C). These results suggest that the AO has therapeutic potential for triple-negative and HER2-overexpressing breast cancers.

Numerous studies have demonstrated that natural compounds exhibit anti-cancer effects by inducing DNA damage and inhibiting DNA repair processes [14]. Increased levels of H2A.X phosphorylation have been implicated in DNA damage [15,16,17,18]. Furthermore, RAD51 is an essential enzyme involved in DNA double-strand break repair through homologous recombination and plays a crucial role in maintaining genomic integrity. Also, RAD51 depletion has been shown to result in the accumulation of DNA damage [19]. Survivin is an inhibitor of the apoptosis protein family and functions as a cell survival factor by inhibiting cell death [20]. Furthermore, it has been reported that survivin depletion impairs homologous recombination-based DNA repair and induces apoptosis [21]. In our study, the AO increased the phosphorylation of H2A.X and reduced RAD51 and survivin levels (Figure 2), which suggests that the AO exhibits cytotoxicity by promoting DNA damage and inhibiting DNA repair.

Generally, DNA damage-induced cytotoxicity is known to induce cell death through the apoptosis pathway [22]. Additionally, DNA damage is recognized to induce apoptosis through the cleavage of PARP via caspase activation [23]. PARP1 is known to function as a substrate of caspase-3 during caspase-dependent apoptosis. Moreover, several reports have indicated that caspase-3 activation in apoptosis occurs early [24,25]. In our results, despite observing an increase in the 89 kDa PARP fragment and late apoptosis on AO treatment for 24 h (Figure 3A,B), caspase-3 activation was not detected. However, we found that caspase-3 cleavage occurred after 2 and 4 h of AO treatment with 45 and 25 µM in MDA-MB-231 and SK-BR-3 cells, respectively (Figure 3C). Moreover, we observed that the cleavage of PARP increased the most after 24 h in both breast cancer cells. Based on these results, we were able to identify a temporal difference between PARP cleavage and caspase-3 activation. These observations suggest that during the cell death process, PARP cleavage is induced by preceding caspase-3 activation, leading to caspase-3-dependent apoptosis. Further investigations are needed to clarify the temporal relationship between caspase activation and PARP1 cleavage.

DNA damage has been reported to induce cell death not only through apoptosis but also via autophagy [26]. Autophagy is a cellular survival process that occurs under stressful conditions, such as nutrient or energy deprivation [27]. However, excessive or dysregulated autophagy can lead to cell death [28]. We observed an interesting phenomenon regarding autophagy markers. Whereas it is commonly reported that autophagy is characterized by an elevation in LC3-II and a suppression in p62 protein levels, we found that the AO increased LC3-II and p62 protein levels (Figure 4A,B). This finding is consistent with several reports indicating that treatment with chloroquine (CQ) and Bafilomycin A1 (a compound that inhibits the fusion between autophagosomes and lysosome) leads to LC3-II and p62 accumulation [29,30,31]. Furthermore, in our results, we found that the protein levels of LC3-II and p62 were still increased by a combined treatment with the AO and CQ (Figure 4C). This finding suggests that despite the inhibition of autophagosome–lysosome fusion by CQ, there is an increase in LC3-II and p62 due to the AO. Therefore, these results indicate that the increase in LC3-II and p62 due to the AO in breast cancer cells is a result of autophagosome accumulation during the autophagy process.

Generally, the inhibition of mTOR phosphorylation increases LC3-II levels and induces autophagy [32]. Some investigations have reported that the mTOR inhibitor rapamycin increases LC3-II and p62 levels [33]. We found that treatment with the AO inhibited the mTOR pathway in MDA-MB-231 and SK-BR-3 cells (Figure 5). Therefore, we suggest that the AO induces autophagy in MDA-MB-231 and SK-BR-3 cells by inhibiting the mTOR pathway and causing LC3-II and p62 accumulation.

The HER2 and EGFR1 signaling pathways exert control over the mTOR pathway via the activation of AKT, a crucial protein kinase involved in cell growth and survival [34,35]. In the present study, the AO inhibited the phosphorylation and protein expressions of EGFR1, HER2, and AKT in SK-BR-3 cells (Figure 6A) and suppressed the protein expression of EGFR1 and phosphorylated AKT in MDA-MB-231 cells (Figure 6B). Lapatinib (a HER2- and EGFR1-targeting agent) and trastuzumab (a HER2-targeting agent) have been reported to hinder the mTOR pathway by inhibiting the phosphorylation AKT [35,36]. Therefore, we suggest that the AO induces autophagy by inhibiting the mTOR pathway by reducing HER2/EGFR1-mediated AKT phosphorylation and EGFR1-mediated AKT phosphorylation in SK-BR-3 and MDA-MB-231 cells, respectively.

**Figure 7:**
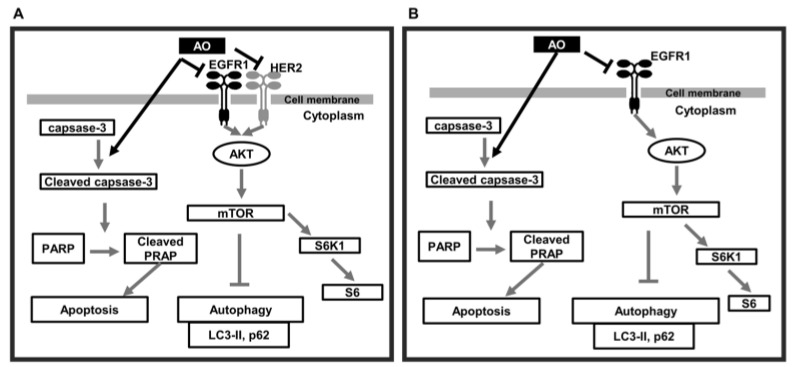
Schematic of the proposed signaling pathways responsible for AO-induced MDA-MB-231 and SK-BR-3 cell death. (**A**) AO induces apoptosis in MDA-MB-231 cells through caspase-3-dependent PARP1 cleavage and activates autophagy by inhibiting the mTOR pathway via EGFR1 and AKT inhibition (**B**) AO induced apoptosis in SK-BR-3 cells through caspase-3-dependent PARP1 cleavage and autophagy induced by inhibiting HER2, EGFR1, and AKT and the mTOR pathway.

In summary, the AO induced DNA damage in MDA-MB-231 and SK-BR-3 cells, and this led to PARP1 cleavage-mediated apoptosis. Furthermore, the AO effectively blocked the mTOR pathway and thus efficiently induced autophagy (Figure 7A,B). The simultaneous occurrence of autophagy and apoptosis in breast cancer cells has been observed. A recently published study has reported that the collaborative effect of autophagy and apoptosis promoted cancer cell death in CRC [37]. Additionally, excessive autophagy has been reported to induce apoptosis in certain research papers [38]. Furthermore, we have previously reported on autophagy-linked apoptosis in breast cancer cells [39,40]. Thus, our findings suggest that the cytotoxic effect of the AO on breast cancer cells is notably enhanced through the collaborative effects of autophagy and apoptosis, demonstrating strong anti-cancer efficacy.

## 4. Materials and Methods

### 4.1. Materials

A synthesized anthocyanin oligomer (AO) was provided as a donation by Kitto Life Co. Ltd. (Pyeongtaek, Republic of Korea) [7,41]. Delphinidin was obtained from Sigma-Aldrich (Merk KGaA, Darmstadt, Germany). Fetal bovine serum (FBS), Dulbecco’s modified eagle medium (DMEM), and antimycotic/antibiotic solution were purchased from Welgene (Daegu, Republic of Korea). Polyvinylidene fluoride (PVDF) membranes were bought from Pall Life Sciences (Port Washington, NY, USA). Protease and phosphatase inhibitor cocktails were from GenDEPOT (Barker, TX, USA). Antibodies for human epidermal growth factor receptor 2 (HER2), phospho-HER2, epidermal growth factor receptor 1 (EGFR1), phospho-EGFR1, protein kinase B (AKT), phospho-AKT (s473), mammalian target of rapamycin (mTOR), phospho-mTOR, ribosomal protein S6 kinase beta-1 (S6K1), phospho-S6K1, ribosomal protein S6 (S6), phospho-S6, microtubule-associated proteins 1A/1B light chain 3 (LC3), phospho-histone 2A.X (p-H2A.X), cleaved caspase-3, poly (ADP-ribose) polymerase 1 (PARP1), and survivin were obtained from Cell Signaling Technology (Beverly, MA, USA). The antibody for p62 (sequestosome-1/SQSTM1) was purchased from ABclonal (Woburn, MA, USA). Antibodies for β-actin, GAPDH, and DNA repair protein RAD51 homolog 1 (RAD51) were from Santa Cruz Biotechnology (Dallas, TX, USA). Additionally, 30% polyacrylamide solution was acquired from SERVA (Heidelberg, Germany). Sulforhodamine B (SRB), Pierce^TM^ BCA Protein Assay Kits, and HRP-conjugated anti-rabbit IgG and -mouse IgG were purchased from Thermo Scientific (Waltham, MA, USA). Prolong^TM^ Gold antifade reagent with DNA stain DAPI and Alexa 488 (Green)-conjugated goat anti-rabbit antibodies were obtained from Invitrogen (Eugene, OR, USA).

### 4.2. Cell Culture

MDA-MB-231 and SK-BR-3 human breast cancer cells were bought from the Korean Cell Line Bank (Seoul, Republic of Korea) and cultured in DMEM supplemented with 1% antibiotic–antimycotic solution and 10% FBS at 37 °C in a 5% CO_2_ incubator. MDA-MB-231 cells are known to be characterized by triple-negative breast cancer, while SK-BR-3 cells are known to be characterized by HER2-overexpressing breast cancer.

### 4.3. Sulforhodamine B (SRB) Cell Viability Assay

MDA-MB-231 and SK-BR-3 cells were seeded in 96-well plates at 3 × 10^3^ and 5 × 10^3^ cells/well, respectively, and permitted to adhere for 24 h. Then, cells were treated with DMEM comprising 4.5, 11.25, 22.5, 45, or 90 μM of the AO or delphinidin (monomeric anthocyanidin) and cultured for an additional 24 h. Cells were then fixed for 1 h at 4 °C in 10% TCA (Trichloroacetic acid), washed four times using tap water, air-dried for 2 h, stained with 1% acetic acid containing 0.4% SRB for 30 min at RT (room temperature), washed four times with 1% acetic acid (without SRB), and air-dried for 2 h. After completely dissolving SRB with 100 µL of 10 mM Tris-HCl (pH 5.0) buffer, absorbances were measured at 510 nm using a Spectra Max M2e (Molecular Devices, Sunnyvale, CA, USA).

### 4.4. Western Blotting

MDA-MB-231 and SK-BR-3 cells were seeded in six-well plates at 3 × 10^3^ and 5 × 10^5^ cells/well, respectively, and allowed to adhere for 24 h. MDA-MB-231 cells were treated with DMEM comprising 4.5, 11.25, 22.5, 45, or 90 µM of the AO for 24 h, whereas SK-BR-3 cells were treated with a conditioned medium containing the AO at 4.5, 11.25, 22.5, or 45 µM for 24 h. In addition, we treated MDA-MB-231 or SK-BR-3 cells with 45 and 22.5 µM of the AO, respectively, for 2, 4, 8, 12, and 24 h. Then, cells were washed twice using cold PBS and lysed by adding 200 µL of RIPA lysis buffer (Biosesang, Seongnam, Republic of Korea) containing 1× phosphatase inhibitor cocktails (sodium fluoride, sodium orthovanadate, sodium pyrophosphate and sodium glycerophosphate) and 1× protease inhibitor cocktails (50 µM PMSF, 10 µM Pepstatin, 20 µM Leupeptin, 100 µM Benzamidine and 50 µM Bestatatin). The lysed cells were centrifuged at 13,000 RPM for 10 min at 4 °C, and the supernatant, excluding the pellet, was transferred to a new microcentrifuge tube and stored at −80 °C until further use. Total protein quantification was carried out using the BCA method, following the protocol provided by the manufacturer. Proteins were separated via SDS-PAGE, transferred onto PVDF membranes, and subsequently blocked with TTBS (0.1% Tween-20, 50 mM Tris-HCl and 150 mM NaCl) supplemented with 1% BSA for 1 h at RT. The membranes were reacted overnight at 4 °C with a primary antibody diluted at 1:3000 in TTBS supplemented with 1% BSA, washed with TTBS, and treated with a secondary antibody diluted at 1:5000 in TTBS for 1 h at RT. Target proteins were visualized by employing a homemade chemiluminescent substrate (100 mM Tris-Cl (pH 8.5), 1.37 mM luminol, 0.01% H_2_O_2_ and 0.22 mM Coumaric acid) and captured using a Luminescent Image Analyzer LAS-4000 (Fujifilm, Tokyo, Japan).

### 4.5. Fluorescent Immunocytochemistry

Cellular expressions of LC3 were assessed by fluorescent immunocytochemistry. MDA-MB-231 and SK-BR-3 cells were seeded in a four-well chamber slide (SPL, Pocheon, Republic of Korea) at 3 × 10^4^ and 5 × 10^4^ cells/well, respectively, and allowed to attach for 24 h. Subsequently, MDA-MB-231 cells were exposed to 45 and 90 µM of the AO, while SK-BR-3 cells were exposed to 22.5 and 45 µM of the AO for 24 h, respectively. Cells were then washed three times with PBS, fixed with ice-cold methanol for 4 min and acetone for 2 min, and washed three times using TTBS for 10 min each. Cells were then blocked with 10% FBS in PBS for 2 h and incubated overnight at 4 °C with primary antibodies for LC3 (1:200 dilution in PBS). Subsequently, cells were washed three times using TTBS for 10 min. Then, cells were incubated for 2 h in the dark at RT with Alexa 488 (Green)-conjugated goat anti-rabbit IgG (1:200 dilution in PBS). Afterwards, cells were exposed toProlong^TM^ Gold antifade reagent with DNA stain DAPI and cover slipped. Cells were photographed, and fluorescence was observed using a fluorescence microscope (Carl Zeiss, Jena, Germany).

### 4.6. Apoptosis Detection through 7-AAD/FITC-Conjugated Annexin V Analysis

MDA-MB-231 and SK-BR-3 cells were seeded at 3 × 10^3^ and 5 × 10^5^ cells/well in six-well plates, respectively. Cells were further cultured for 24 h to promote attachment. MDA-MB-231 cells were exposed to DMEM comprising 22.5 µM, 45 µM, or 90 µM of the AO for 24 h, whereas SK-BR-3 cells were exposed to a conditioned medium containing the AO at 11.25 µM, 22.5 µM, or 45 µM for 24 h. Cell harvesting was performed using trypsinization. The cells were stained using an FITC Annexin V Apoptosis Detection Kit with 7-AAD (BioLegend, San Diego, CA, USA) following the manufacturer’s protocol. Following staining, cell analysis was performed using an FACS Calibur II and Cell Quest Pro software (version 5.2.1, BD Biosciences, San Jose, CA, USA).

### 4.7. Statistical Analysis

The reported data consisted of the mean ± standard error of the mean (SEM) obtained from three independent experiments, each conducted three times. The significance of the differences was assessed using one-way ANOVA, with AO treatment concentrations as the independent variable and the relative intensity of each protein as the dependent variable. Fisher’s least significant difference (LSD) tests were conducted to compare the untreated (0 uM) group with different treatment groups, determining significant differences between each treatment group and the untreated group. The analysis was conducted using SPSS Ver. 20.0 software (SPSS, Inc., Chicago, IL, USA), and results are reported as means with their corresponding standard deviations (SDs). To represent statistical significance on each graph, * was used when *p* was less than 0.05, and ** was used when *p* was less than 0.01.

## 5. Conclusions

In conclusion, this study highlights the potential use of AOs for the treatment of HER2-overexpressing and triple-negative breast cancer. The effects of a synthetic AO on SK-BR-3 (HER2-overexpressing) and MDA-MB-231 (triple-negative) breast cancer cells were found to be mediated by apoptosis and autophagy. However, here we report the anti-cancer effect of the AO at a cellular level, which means animal experiments are required to evaluate the potential clinical efficacy of AOs as a treatment for breast cancer.

## Data Availability

Data are contained within the article and Supplementary Materials.

## Supplementary Materials

The following supporting information can be downloaded at: https://www.mdpi.com/article/10.3390/ph17010024/s1, Figure S1: The comparative evaluation of the AO effect on cell viability over 24, 48, and 72 hours using the SRB assay in MDA-MB-231 and SK-BR-3 cells.
