# Supplementary material for: Anthocyanin Oligomers Induce Apoptosis and Autophagy by Inhibiting the mTOR Signaling Pathway in Human Breast Cancer Cells
Source: Pharmaceuticals (Basel). 2023 Dec 22;17(1):24. doi: 10.3390/ph17010024 (PMC10820553; doi:10.3390/ph17010024)
Supplement: Supplementary file 1 [file pharmaceuticals-17-00024-s001.zip › pharmaceuticals-2745319-supplementary.pdf]

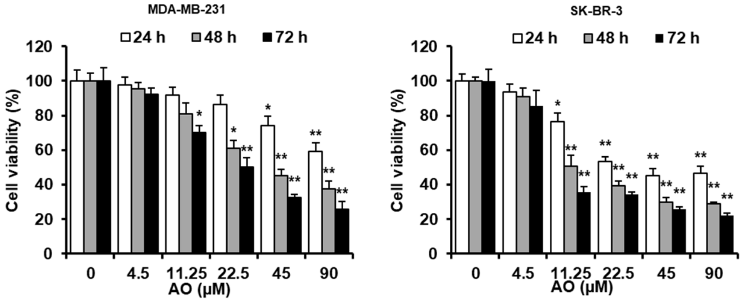

Figure S1. The comparative evaluation of the AO effect on cell viability over 24, 48, and 72 hours using the SRB assay in MDA-MB-231 and SK-BR-3 cells. AO exhibited a concentration- and time-dependent decrease in cell viability in breast cancer cells. Results are presented as means ± SDs (n=8). Statistical significance is indicated by * representing p-values below 0.05, while ** represents those below 0.01. The Statistical differences were assessed using one-way analysis of variance (ANOVA) and subsequently verified using Fisher's least significant difference (LSD) test.
